# Three-year clinical evaluation of zirconia and zirconia-reinforced lithium silicate crowns with minimally invasive vertical preparation technique

**DOI:** 10.1007/s00784-022-04779-1

**Published:** 2022-11-16

**Authors:** Ammar T. Kasem, Mohamed Ellayeh, Mutlu Özcan, Amal Abdelsamad Sakrana

**Affiliations:** 1 Fixed Prosthodontics Department, Faculty of Dentistry, Mansoura University, Mansoura, Egypt; 2 Prosthodontics Department, Faculty of Dentistry, New Mansoura University, New Mansoura, Egypt; 3 Division of Dental Biomaterials, Center for Dental and Oral Medicine, University of Zurich, Clinic for Reconstructive Dentistry, Zurich, Switzerland; 4 Fixed Prosthodontics Department, Faculty of Dentistry, Horus University, New Damietta, Egypt

**Keywords:** Vertical preparation, Biologically oriented preparation technique, Minimal invasive, Zirconia-reinforced lithium silicate, FDI criteria

## Abstract

**Objectives:**

Large part of the tooth is required to be removed during crown preparation. A minimally invasive method for preparing single crowns is required to increase the durability of teeth. The aim of this study was to evaluate the clinical performance of two ceramic systems fabricated with minimally invasive vertical preparation.

**Materials and methods:**

Forty endodontically treated maxillary premolars were prepared with vertical preparation and received temporary crowns for a period of 21 days. Twenty zirconia-reinforced lithium silicate (Celtra Duo HT, Dentsply Sirona, Germany) and 20 monolithic high translucency zirconia (Katana HT, Kuarary Noritake, Japan) crowns were fabricated by CAD/CAM and cemented with dual-polymerizing luting resin. The crowns were evaluated clinically and radiographically for 36 months following modified FDI criteria. Statistical analysis was conducted with *t* Student test (Cochran *Q*).

**Results:**

Over the follow-up period, there was no need to replace any of the study’s crowns. The overall survival rate of the 40 crowns was 100% according to the Kaplan–Meier survival method. The clinical quality of all crowns and the patient’s satisfaction were high. No caries was detected and no adverse soft tissue reactions around the crowns were observed. Periodontal probing depth was reported to be increased at mesial and distal sites more than the facial one in the 36-month follow-up with no statistically significant difference between both materials (*P* = 0.186).

**Conclusions:**

Zirconia and zirconia-reinforced lithium silicate could be used as a material for restoration of teeth prepared with vertical preparation technique. Both ceramic materials achieved good esthetic results, promotes healthy and stable soft tissues with no mechanical complications after 3 years of clinical evaluation.

**Clinical relevance:**

Monolithic high translucency zirconia and zirconia-reinforced lithium silicate ceramics can be used for the restorations of minimal invasive vertical preparation in premolar area with 0.5 mm margin thickness.

## Introduction

The primary challenge of restorative dentistry is how to obtain an excellent esthetic outcome while preserving tooth structure through minimally invasive preparation techniques [1, 2]. Minimizing tooth preparation promotes the preservation of the tooth structure and dento-enamel junction, which has a significant role in the redistribution of stresses, resisting cracks propagation, and increasing the durability of teeth [3, 4].

Two types of dental preparation are well known in the literature. The first type is horizontal preparation with a well-defined finish line, which is then replicated in the impression and the working model. The second type is preparation without a finish line, also known as vertical preparation or feather edge [5, 6]. For vertical preparation, the margins are determined by the laboratory technician based on gingival tissue information [7]. Vertical preparations are commonly indicated for periodontally involved teeth that serve as abutments for fixed dental prosthesis (FDP). Periodontally involved teeth which are usually associated with observed gingival recession require the removal of substantial amount of tooth structure to achieve a horizontal finish line that possibly compromises the long-term prognosis of the teeth [8, 9]. Vertical preparation offers a conservative alternative, where the finish line is represented by an area rather than a horizontal line [10, 11].

Recently, the utilization of vertical preparation with a biologically oriented preparation technique (BOPT) has been suggested [12, 13]. BOPT is a protocol in which a new prosthetic cemento-enamel junction is created to replace the anatomical crown’s emergence profile corresponding to the cemento-enamel junction. A key factor for a successful BOPT protocol is fabricating an optimal interim prosthesis that determines the new prosthetic emergence profile which will support the gingival margin and guide the healing and thickening of the gingival tissue. This will be reproduced when the definitive prosthesis is placed [14, 15].

Three main factors should be considered to achieve an optimal dental restoration; resistance to fracture, marginal adaptation, and esthetic value [11, 16]. Recently, two all-ceramic monolithic dental materials are mainly used in most restorative conditions for their ideal mechanical and esthetic characteristics; glass ceramics, and polycrystalline zirconium dioxide [17, 18]. The noble mechanical properties of zirconia allow clinicians to adopt changes in the preparation strategies concerning the coping design [19, 20]. This can allow reducing the coping thickness from 0.5 to 0.3 mm and changing the chamfer finish line to the more conservative feather-edge design [21, 22]. It has been shown that restorations manufactured from yttria-stabilized tetragonal zirconia polycrystal (Y-TZP) may provide clinical longevity of 20 years [23, 24].

Recently, monolithic glass–ceramic materials are developed to provide exceptional esthetics avoiding the drawbacks of layered ceramics [25, 26]. Zirconia-reinforced lithium silicate (ZLS) glass ceramics are now widely used as machinable ceramics for CAD/CAM techniques [27]. These materials offer flexural strength varying from 370 to 420 MPa which is comparable with the lithium disilicate glass ceramics. Moreover, it offers an improved esthetic outcome and bond strength compared to that of zirconia ceramics [28].

The amount of tooth preparation required for most traditional all-ceramic systems varies from 1.2 to 1.5 mm axially and 1.5 to 2 mm occlusally which equal that of porcelain fused to metal restorations [29, 30]. Advances in mechanical properties and improved optical characteristics of current all-ceramic systems enabled the use of more conservative and less invasive preparation designs [3, 4]. Zirconia and ZLS ceramics with minimal invasive vertical preparation showed a comparable performance between conventional and vertical preparations [18]. Based on the results of this evaluation, this clinical study was performed to evaluate the performance of zirconia and ZLS ceramics with minimal invasive vertical preparation technique according to modified FDI criteria for 36-month follow-up period.

## Materials and methods

### Study design

A total of 40 crowns (*n* = 40) were placed for 24 patients who presented for restoration of endodontically treated maxillary premolar teeth. Patients’ selection was based on a set of inclusion and exclusion criteria (Table 1) [31]. The patients were selected from the outpatient dental clinic, Faculty of Dentistry, Mansoura University, Egypt. This clinical study was performed in accordance with the guidelines of the Declaration of Helsinki [32]. The research protocol was approved by Dental Research Ethics Committee in Mansoura University (05,060,218/2018), prior to patient enrollment. Treatment options were explained to the patients in terms of steps, benefits, and risks then a written consent was signed by each patient indicating his/her willingness to be part of this study. After a screening appointment to verify patient eligibility, the teeth planned for crowns were randomly assigned to the study groups according to the material used. The information about the study participants and groups is shown in (Table 2).

**Table 1:** Inclusion and exclusion criteria of this study

Inclusion criteria	Exclusion criteria
(1) Healthy patients in the age range from 20 to 40 years (2) Good oral hygiene (3) Good quality of root canal treatment with no periapical lesions (4) Periodontal probing depth prior to tooth preparation ≤ 2 mm and no bleeding on probing (5) > 2 mm of keratinized tissue (6) There are a minimum three remaining adjacent sound dentin walls with composite core foundation (7) The patients should be available during the follow-up schedules	(1) Smoking (> 10 cigarettes/day) (2) Any local or systemic disease or medication that might compromise healing and affect the periodontium (3) Inability to give informed consent to participate in this study (4) History of alcohol or drug abuse (5) Unfavorable crown-to-root ratio (6) Severe clenching or bruxing habits

**Table 2:** Participants and groups features

	Group ZLS	Group Z
Restoration type	Zirconia-reinforced	Monolithic zirconia crowns
	lithium silicate crowns	
Brand name	Celtra Duo	Katana
Manufacturer	Dentsply Sirona, Germany	Kuarary Nortikate, Japan
Translucency	High translucency	High translucency
Flexural strength	370 MPa after glazing*	1125 MPa after sintering*
Number of crowns	20	20
Total participants	10	14
Sex (male/female)	4/6	6/8
Age	22–36	22–38
Missed during Follow-up period	2	1
	Replaced with another participants	
Replaced crowns	1	0

^*^According to the data from the manufacturers

### Prosthetic procedures

**Fig. 1:**
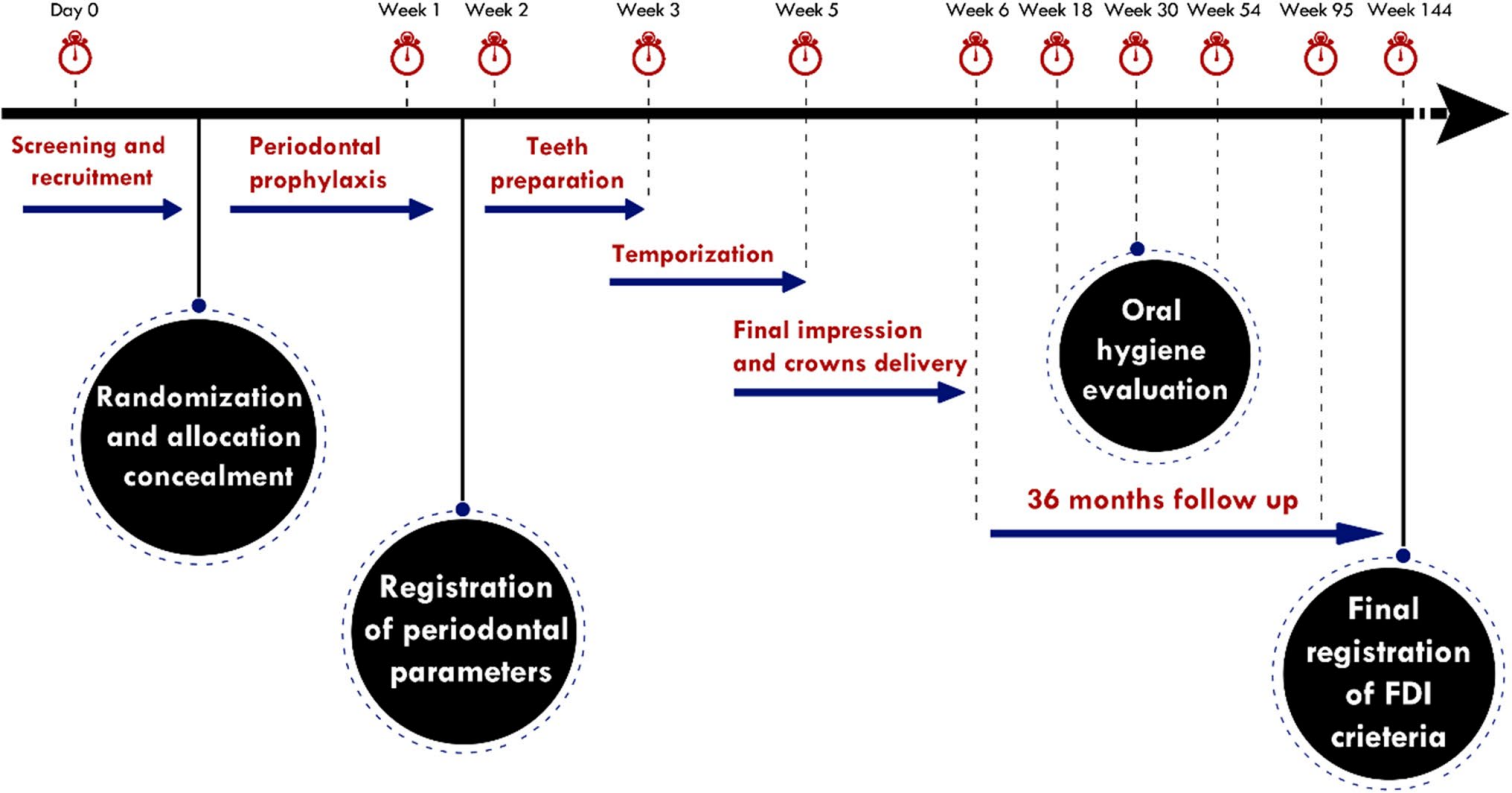
Diagram showing the time sequence of interventions and examinations

The time sequence of all procedures is shown in (Fig. 1). One week before preparation, all patients underwent scaling and prophylaxis to enhance the gingival condition. Then, the periodontal probing depth (PPD) was registered at three facial and palatal sites (mesial, midpoint, distal) and also proximally using a periodontal probe rounding the measurements to the nearest millimeter.

The shade selection procedure was done for all selected patients before preparation using both visual and instrumental methods to complement each other for the accurate esthetic outcomes. The conventional visual method for shade selection was performed using the IVOCLAR VIVADENT A-D shade guide (Ivoclar Vivadent, Liechtenstein). Then, a portable clinical spectrophotometer (Vita Easyshade V, Vita Zahnfabrik, Germany) was used to identify digitally and precisely the patient teeth shade required for the shade selection of the crowns.

All teeth preparations were carried out by one prosthodontist under local anesthesia (articaine with 1:100.000 epinephrine). The standardized technical procedures were performed using a kit for vertical preparation (0197, Komet, Germany). A minimal invasive preparation for all-ceramic crown was carried out, with an initial depth of 0.8 mm to a final depth of 1 mm axially and 0.5 mm at the marginal area. The occlusal surface was reduced by 1.5 mm with a 1-mm minimal thickness in the thinnest portion of the crown.

**Fig. 2:**
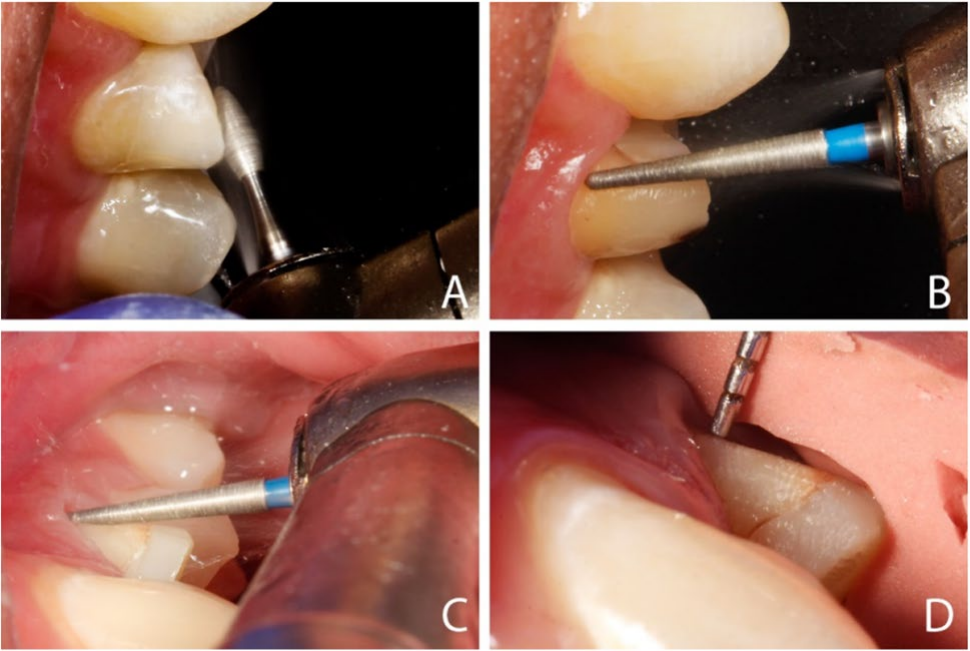
Steps of vertical preparation**. A** 1.5-mm occlusal reduction. **B** 0.8-mm initial axial reduction of supra-gingival part with chamfer preparation using tapered diamond stone with round end. **C** 0.5–1 mm sub-gingival preparation. **D** Checking the preparation by putty index

**Fig. 3:**
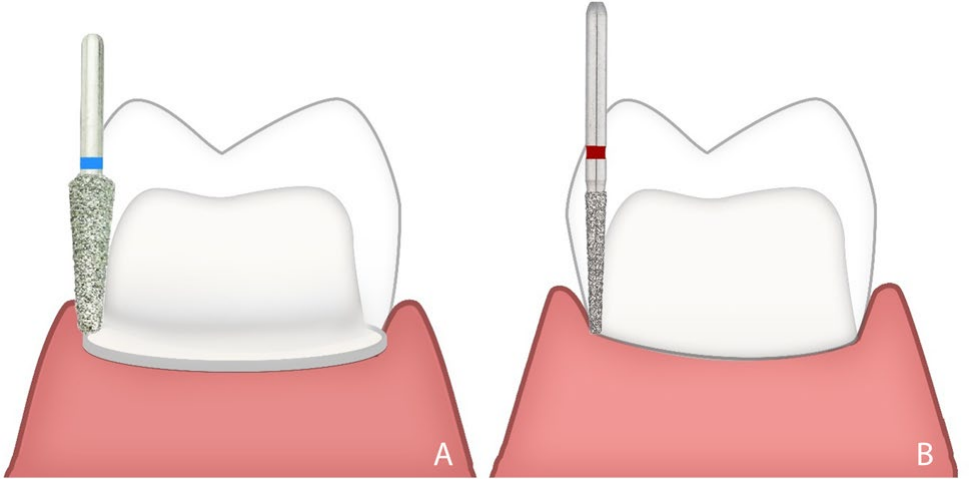
Illustration showing the difference between **A** conventional and **B** vertical preparations

A rotary curettage of the gingival sulcus (gingitage) was performed simultaneously with vertical tooth preparation [33]. To avoid any violation of the biologic width, the preparation extended sub-gingivally not deeper than 1 mm from the gingival margin using a 4 × magnification loupe (Wenzhou Amtech Medical Technology Co., China) regardless of the probing depth recorded at baseline. Teeth were prepared with a vertical margin (Fig. 2), so there was a finishing area rather than a finish line. The reduction was started by reducing the occlusal surface by 1.5 mm using a football stone. Fine-tapered stone was then used to separate the interproximal contact area. The axial reduction was started using a tapered diamond with rounded end to create a supra-gingival chamfer finishing line. Using the batt bur (857–314-014, Komet, Germany), the preparation was extended sub-gingival removing the formerly prepared finish line. The preparation was extended 0.5–1 mm apical to the free gingiva with a 6 to10° convergence angle. The preparation was then checked by putty index to make standardization of preparation. The difference between vertical and conventional preparation is shown in (Fig. 3).

**Fig. 4:**
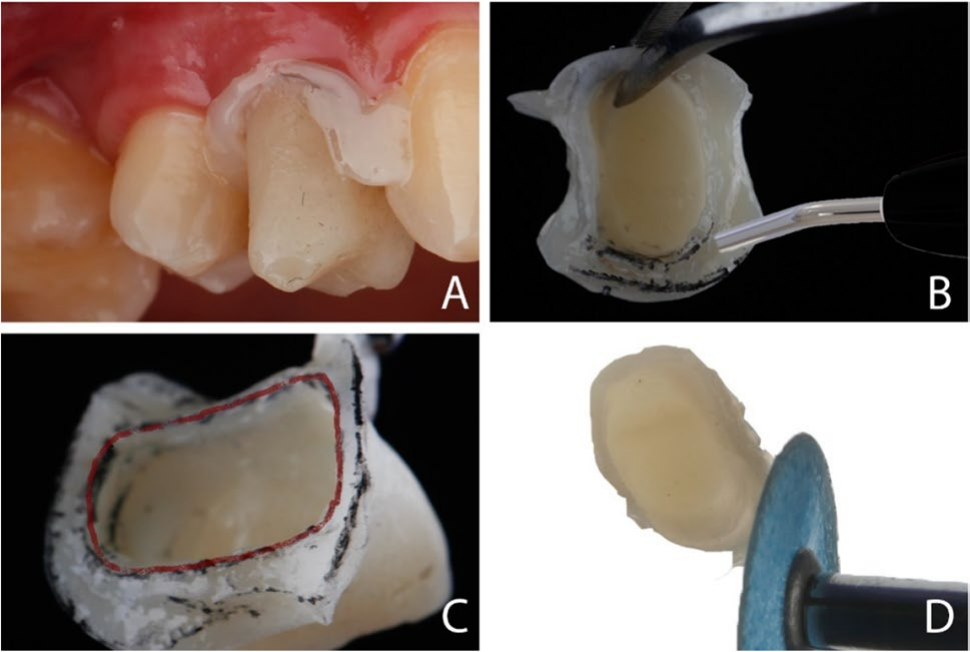
Photographs showing relining of the temporary crown. **A** Relined temporary crown was inserted in patient mouth. **B** The space between the two margins was filled by flowable composite. **C** The internal margin was evidenced by red marker. **D** The excess resin was trimmed by discs and the emergence profile was shaped to support the gingival margin

The direct temporization technique followed by relining was used. After fit verification, all crowns were relined using auto-polymerizing resin (VISALYS TEMP, Kettenbach GmbH & Co.kg, Germany) [7]. Two distinct margins for the crown were shown after the setting of temporary material; a thin internal margin, which replicates the intra-sulcular part of the preparation, while the external one was thicker which follows the external portion of marginal gingiva. The space between both margins represents the negative image of the gingiva (Fig. 4). After that, a light-polymerized flowable composite was used to fill the space between the two margins to make the coronal margin thicker and create the crown contour. In this way, a new CEJ was obtained in the sulcus not deeper than 0.5 to 1 mm, respecting the tooth biologic width. After an accurate finishing with flexible aluminum oxide discs (3 M™ Sof-Lex™ Discs) and polishing with diamond wheels (3 M™ Sof-Lex™ Diamond Spirals), the crown was cemented using provisional cement (Provilat, Promedica, Germany).

**Fig. 5:**
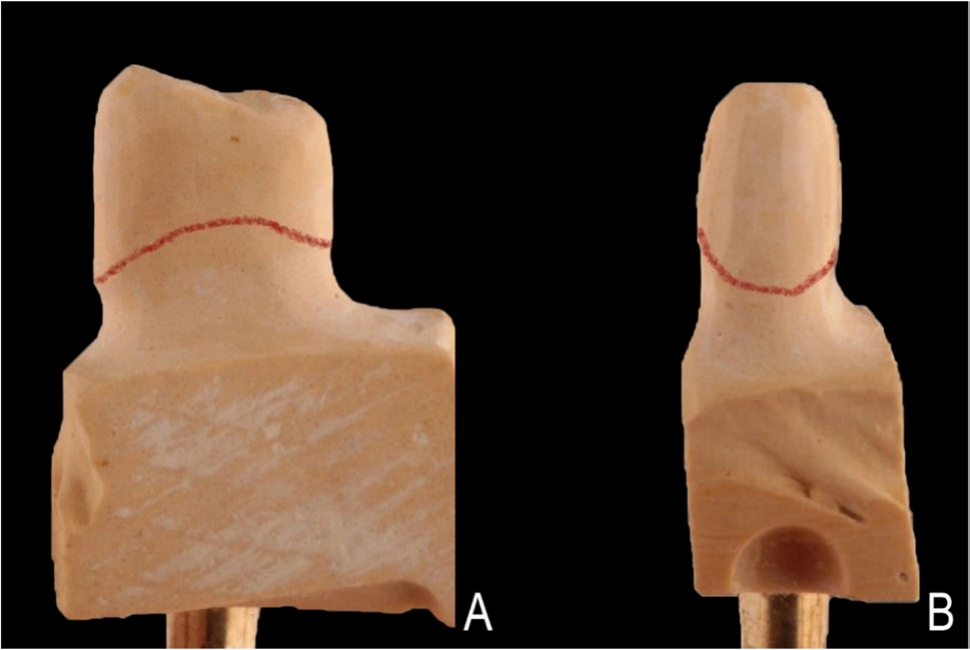
Photographs showing removable die after preparation and ditching. **A** Bucco-lingually and **B** mesio-distally

Patients were instructed to use Chlorhexidine gluconate solution (0.2%) for 7 days until they could practice regular oral hygiene. The temporary crowns were left in place for a period of 14–21 days [7, 12] to promote the healing process and allow for the thickening of gingival tissue. After the period of temporization, the gingival tissue became stabilized and final impressions were taken using two-step polyvinyl siloxane impression material (Elite HD + putty soft, Zhermack, Italy). Double retraction cords were applied for 5 min before recording the impression using two different cord sizes #00 and #1 cord (Gingicord, Denu, Korea) impregnated with a hemostatic agent (aluminum chloride). The impression was poured twice, the first cast was used for ditching and scanning and the other one was used for checking and verification of the crowns before insertion. The gingival part around the abutment was removed showing the sub-gingival area of the preparation reproduced on the model as shown in (Fig. 5).

The casts were scanned using a 3D optical scanner (Ceramill Map400, AmannGirrbach, Germany) and designed with compatible software (Ceramill Mind CAD, version 3.5.6.1408, AmannGirrbach GmbH, Germany). Twenty high translucency ZLS crowns were wet milled and 20 monolithic high translucency zirconia crowns were dry milled using a CAM system (Ceramill motion II, AmannGirrbach, Germany). For ZLS, the crowns were subjected to a first glaze firing process at 820 C, then additional glaze firing was performed at 770 C (heating rate 55 C /min, hold time 1:30 min). The sintering of zirconia crowns was performed by raising the temperature of the ceramic furnace from room temperature to 1450 C for 2.5 h and holding this temperature for 2 h, and then decreasing it to room temperature again for 2.5 h.

The crowns of ZLS group were etched using 9.5% hydrofluoric acid for 20 s according to the manufacturer’s instructions. Then, acid residues were removed from the crowns intaglio surface by rinsing under running water. Crowns were dried using air syringe till the appearance of chalky white surface. Finally, one coat of porcelain primer was applied to the intaglio surface, and then air-dried for 3–5 s following the manufacturer’s instructions. For group Z, the intaglio surface was air borne particle abraded using Al_2_O_3_ particles (50 µm; 4 bar (0.4 MPa) air pressure for 14 se). The tip of the sandblaster device was adjusted 10 mm away from the crowns [34]. Then crowns’ intaglio surfaces were covered by one coat of zirconia primer, then air-dried for 3–5 s following the manufacturer’s instructions. Since the margins were placed inside the intra-sulcular compartment, alternative methods rather than rubber dam were used to isolate the environment from moisture, such as retractors for the lips, cotton rolls, and retraction cords to control the sulcular fluids and to remove excess cement sub-gingivally [35]. Dual-polymerizing self-adhesive universal luting resin (G-CEM Capsules, GC Co., Japan) was used for the cementation of all crowns of both groups.

### Clinical follow-up protocol

Clinical follow-up was stated 48 h after cementation (baseline) then 6, 12, 18, 24, and finally after 36 months [36]. At each recall appointment, both clinical examinations and periapical radiographs were performed using modified FDI criteria [37]. Clinical examinations were performed using a mirror, sharp explorer, and digital photographs then the final score was taken based on a 3-examiner evaluation. The new FDI criteria set a different background for the evaluation of dental restorations by introducing 3 groups of criteria; esthetic, functional, and biological. Each of these groups has subgroups with the final score in each group being dictated by the most severe score among all the sub-scores. The selected FDI criteria and the methods of evaluation for each criterion are described in (Table 3).

**Table 3:** FDI criteria and the method of evaluation for each selected criterion

Criteria	Method of evaluation
Surface luster	- A qualitative inspection in relation to neighboring enamel with thoroughly cleaned and dried restored tooth and switched off operator light at a distance of 60–100 cm
Staining	- Clinical inspection using mirror and illumination
Color match and translucency	- Comparison to that of the surrounding tooth tissue and adjacent teeth using visual and instrumental (Vita Easyshade V) methods - A standard set of digital photographs were also provided for stability comparison
Esthetic anatomical form	- Visual comparison to the normal form with switched off operator light at a distance of 60–100 cm
Fracture of material and retention	- Clinical inspection using mirror/probe, loupe magnification, and proper illumination after thorough cleaning and dryness
Occlusal contour and wear	- Qualitatively through photo-documentation of the occlusal surface (contact areas) of crowns, antagonist, and adjacent teeth (reference enamel) at baseline and recall appointments
Approximal anatomical form	- Approximal contact points were evaluated with metal blades/strips (25, 50, and 100) (TOR VM Ltd, Moscow, Russia). Waxed dental floss was used for calibration at baseline and at all recalls - Approximal contour was evaluated through visual assessment with regard to the normal
Radiographic examination	- X-rays for the involved teeth at each recall appointment
Patient view	- Structured interview with the patient on his/her satisfaction/dissatisfaction with the crown
Tooth integrity	- Clinical inspection using mirror/probe, loupe magnification, and proper illumination after dryness - A set of blunt probes, straight, and double angled for proximal sites, with different blunt tips of 50, 150, and 250 μm were used (DENTSPLY Maillefer Instruments, Ballaigues, Switzerland)
Periodontal response	- Clinical inspection of the involved tooth using mirror, periodontal probe, and papillary bleeding index (PBI scale 0–4) with comparison to a control reference tooth
Oral and general health	- Broad clinical inspection of the oral cavity and the medical status and history of the patient

### Statistical analysis

The data were tabulated, coded, then analyzed in the environment of IBM SPSS (Statistical package for social sciences) computer software, version 23.0. Descriptive statistics were expressed as mean (SD) and valid percentages for continuous and categorical data. The baseline comparisons and continuous variables between groups were performed using *t* Student test (Cochran *Q*).

## Results

Twenty-four patients (10 males and 14 females, aged 22–38 years, mean age 30 years) received 40 crowns of which 20 were included in group ZLS and 10 in group Z. All participants completed the 36-month follow-up period except for 2 patients related to group ZLS and 1 patient related to group Z were missed and replaced by other participants. Only one crown in group ZLS was fractured during insertion and before cementation due to extra load from the patient, and the fractured crown was replaced with a new one. Regarding group Z, one crown was debonded after 30 months and re-cemented.

**Fig. 6:**
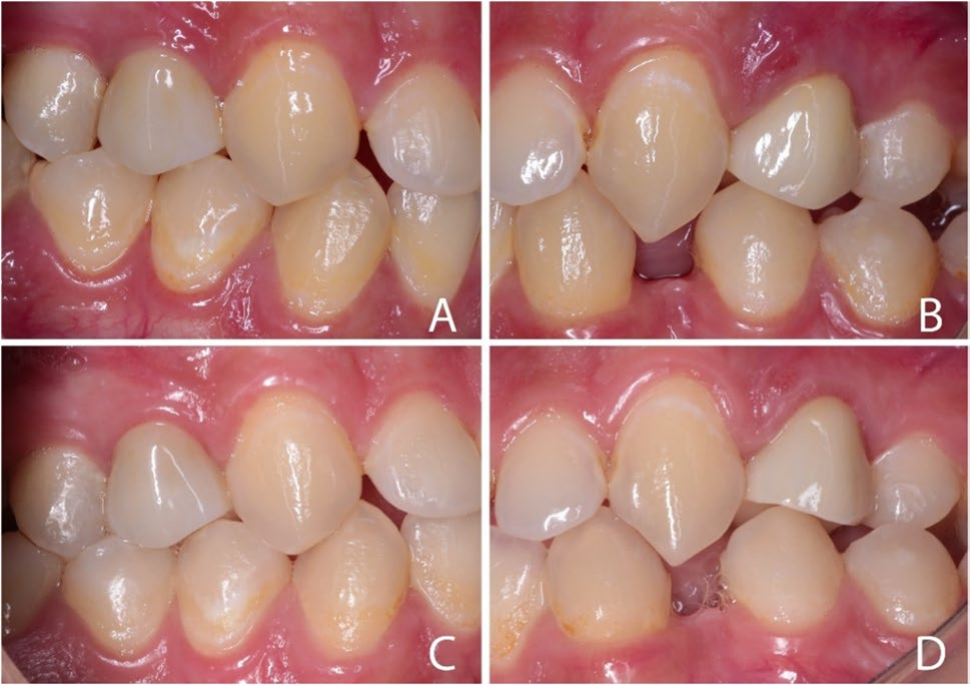
Intra oral photographs: showing **A** Celtra Duo and **B** KATANA crowns 48 ho after cementation. **C** Celtra Duo and **D** KATANA crowns at 36-month follow-up appointment

**Fig. 7:**
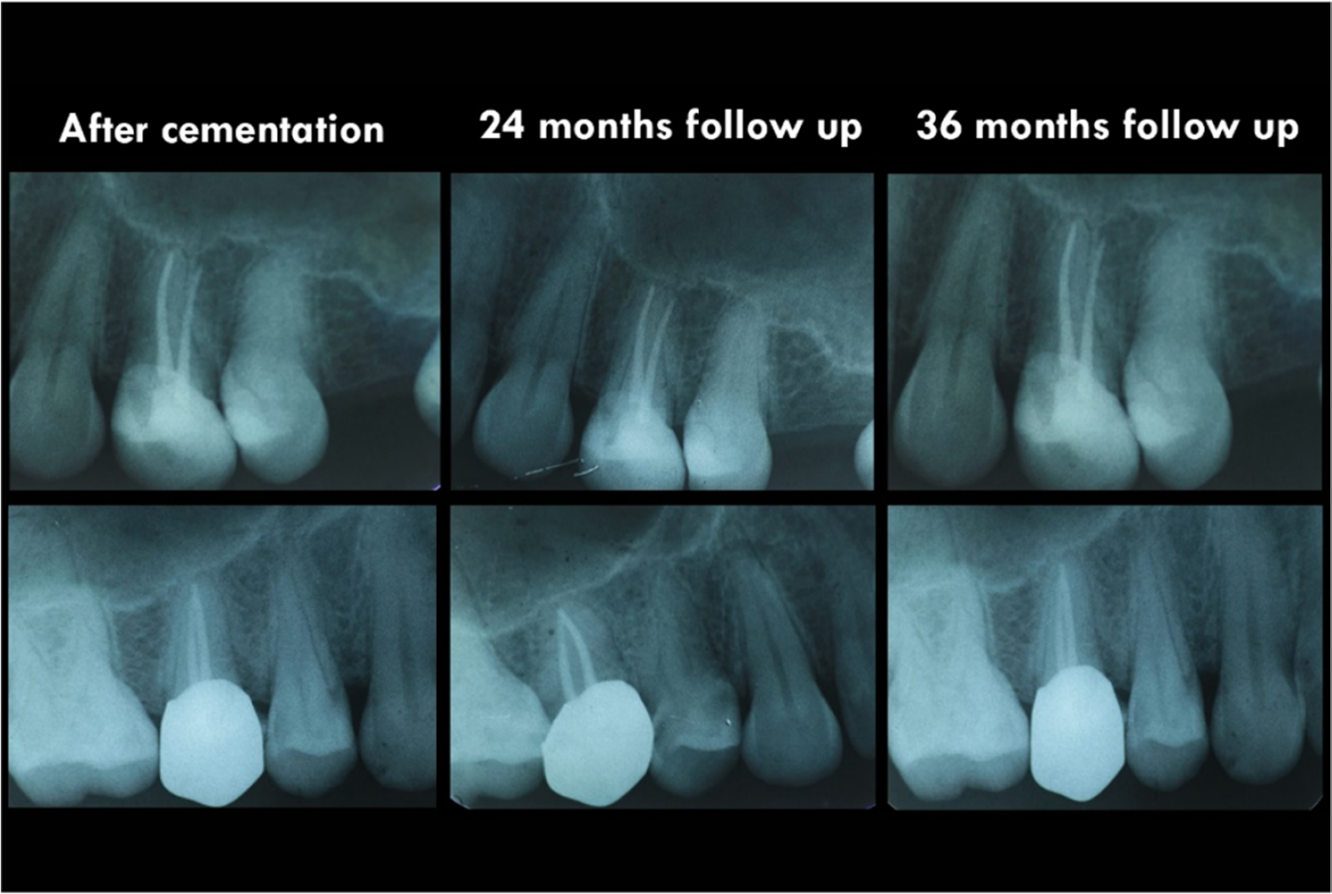
Radiographic images: showing Celtra Duo (above) and KATANA (below) crowns during follow-up period. No pathology, no pockets, and there was a harmonious transition between crowns and neighboring teeth

All crowns were evaluated clinically and radiographically at baseline (48 h after cementation), 6, 12, 18, 24, and finally at 36 months. Clinical evaluation was done using diagnostic tools and digital photographs (Figs. 6 and 7). All scores were drawn after observation by the same clinicians. Scores 3, 4, and 5 were not observed through the evaluation period for all the tested criteria so, they were excluded from the results. Over the follow-up period, there was no need to replace any of the study’s crowns. The overall survival rate of the 40 crowns was 100% according to the Kaplan–Meier survival method [38]. All patients were satisfied with esthetic and functional outcomes at all examinations.

### Esthetic properties (Tables 4)

**Table 4:** Esthetic properties among studied groups with difference between follow-up scores

Esthetic properties	Group	FDI score (1–5) ∗	Baseline *N* (%)	12 months *N* (%)	24 months *N* (%)	36 months *N* (%)	Test of significance (Cochran *Q*)
Surface luster	ZLS	Score 1	20 (100.0)	20 (100.0)	20 (100.0)	20 (100.0)	**…**
		Score 2	0 (0.0)	0 (0.0)	0 (0.0)	0 (0.0)	
	Z	Score 1	20 (100.0)	20 (100.0)	16 (80.0)	16 (80.0)	*P* = 0.007*
		Score 2	0 (0.0)	0 (0.0)	4 (20.0)	4 (20.0)	
Test of significance			**…**	**…**	*P* = 0.106	*P* = 0.106	
Surface staining	ZLS	Score 1	20 (100.0)	20 (100.0)	18 (90.0)	16 (80.0)	*P* = 0.047*
		Score 2	0 (0.0)	0 (0.0)	2 (10.0)	4 (20.0)	
	Z	Score 1	20 (100.0)	20 (100.0)	10 (50.0)	6 (30.0)	*P* < 0.001*
		Score 2	0 (0.0)	0 (0.0)	10 (50.0)	14 (70.0)	
Test of significance			**…**	**…**	*P* = 0.661	*P* = 0.333	
Color match and translucency	ZLS	Score 1	14 (70.0)	14 (70.0)	14 (70.0)	14 (70.0)	*P* = 1.0
		Score 2	6 (30.0)	6 (30.0)	6 (30.0)	6 (30.0)	
	Z	Score 1	18 (90.0)	18 (90.0)	18 (90.0)	18 (90.0)	*P* = 1.0
		Score 2	2 (10.0)	2 (10.0)	2 (10.0)	2 (10.0)	
Test of significance			*P* = 0.114	*P* = 0.114	*P* = 0.114	*P* = 0.114	
Esthetic anatomical form	ZLS	Score 1	18 (90.0)	18 (90.0)	18 (90.0)	18 (90.0)	*P* = 1.0
		Score 2	2 (10.0)	2 (10.0)	2 (10.0)	2 (10.0)	
	Z	Score 1	20 (100.0)	20 (100.0)	20 (100.0)	20 (100.0)	*P* = 1.0
		Score 2	0 (0.0)	0 (0.0)	0 (0.0)	0 (0.0)	
Test of significance			*P* = 0.487	*P* = 0.487	*P* = 0.487	*P* = 0.487	

∗Score: 1 = clinically excellent/very good, 2 = clinically good, 3 = clinically sufficient/satisfactory, 4 = clinically unsatisfactory (but repairable), 5 = clinically poor (replacement necessary)

Regarding group ZLS, the surface luster was not changed for all crowns over the evaluation period and was scored as 1; however, four crowns belonging to group Z were scored as 2 in the 24- and 36-month control appointments. Surface staining did not change over 6 months and was scored as 1 for both groups; however, two crowns in group ZLS was scored as 2 at 12 months and four crowns were scored as 2 at 24- and 36-month control appointments. For group Z, ten crowns were scored as 2 at the 12-month control appointment and 14 crowns were scored as 2 at the 24- and 36-month control visits. Color match and translucency was scored as 1 for all crowns of both groups during the 36-month follow-up period except for six crowns in group ZLS and two crowns in group Z were scored 2 from the baseline. The esthetic anatomical form did not change during the evaluation period and was scored as 1 for all crowns of both groups except for two crowns belonging to group ZLS was scored as 2 from the baseline.

### Functional properties (Tables 5)

**Table 5:** Functional properties among studied groups with difference between follow-up scores

Functional properties	Group	FDI score (1–5) ∗	Baseline *N* (%)	12 months *N* (%)	24 months *N* (%)	36 months *N* (%)	Test of significance (Cochran *Q*)
Fracture of material and retention	ZLS	Score 1	20 (100.0)	20 (100.0)	20 (100.0)	20 (100.0)	**…**
		Score 2	0 (0.0)	0 (0.0)	0 (0.0)	0 (0.0)	
	Z	Score 1	20 (100.0)	20 (100.0)	20 (100.0)	20 (100.0)	**…**
		Score 2	0 (0.0)	0 (0.0)	0 (0.0)	0 (0.0)	
Test of significance			**…**	**…**	**…**	**…**	**…**
Occlusal wear	ZLS	Score 1	20 (100.0)	20 (100.0)	20 (100.0)	20 (100.0)	**…**
		Score 2	0 (0.0)	0 (0.0)	0 (0.0)	0 (0.0)	
	Z	Score 1	20 (100.0)	20 (100.0)	20 (100.0)	16 (80.0)	*P* = 0.005*
		Score 2	0 (0.0)	0 (0.0)	0 (0.0)	4 (20.0)	
Test of significance			**…**	**…**	**…**	*P* = 0.106	
Approximal anatomical form	ZLS	Score 1	20 (100.0)	20 (100.0)	20 (100.0)	20 (100.0)	**…**
		Score 2	0 (0.0)	0 (0.0)	0 (0.0)	0 (0.0)	
	Z	Score 1	20 (100.0)	20 (100.0)	20 (100.0)	18 (90.0)	*P* = 0.104
		Score 2	0 (0.0)	0 (0.0)	0 (0.0)	2 (10.0)	
Test of significance			**…**	**…**	**…**	*P* = 1.0	
Radiographic examination	ZLS	Score 1	20 (100.0)	20 (100.0)	20 (100.0)	20 (100.0)	**…**
		Score 2	0 (0.0)	0 (0.0)	0 (0.0)	0 (0.0)	
	Z	Score 1	20 (100.0)	20 (100.0)	20 (100.0)	20 (100.0)	**…**
		Score 2	0 (0.0)	0 (0.0)	0 (0.0)	0 (0.0)	
Test of significance			**…**	**…**	**…**	**…**	
Patient view	ZLS	Score 1	20 (100.0)	20 (100.0)	20 (100.0)	20 (100.0)	**…**
		Score 2	0 (0.0)	0 (0.0)	0 (0.0)	0 (0.0)	
	Z	Score 1	20 (100.0)	20 (100.0)	20 (100.0)	20 (100.0)	**…**
		Score 2	0 (0.0)	0 (0.0)	0 (0.0)	0 (0.0)	
Test of significance			**…**	**…**	**…**	**…**	

∗Score: 1 = clinically excellent/very good, 2 = clinically good, 3 = clinically sufficient/satisfactory, 4 = clinically unsatisfactory (but repairable), 5 = clinically poor (replacement necessary)

Fracture of material and retention, radiographic examination, and patient’s view were scored as 1 during the clinical evaluation period for all crowns in both groups. Approximal anatomical form also was scored as 1 for all ZLS crowns during the follow-up period. Regarding group Z, approximal anatomical form was scored as 1 for all crowns during the first year and only two crowns were scored 2 in the 24- and 36-month follow-up appointments.

### Biologic properties (Tables 6)

**Table 6:** Biological properties among studied groups with difference between follow-up scores

Biological properties	Group	FDI score (1–5) ∗	Baseline *N* (%)	12 months *N* (%)	24 months *N* (%)	36 months *N* (%)	Test of significance (Cochran *Q*)
Tooth integrity	ZLS	Score 1	20 (100.0)	20 (100.0)	20 (100.0)	20 (100.0)	**…**
		Score 2	0 (0.0)	0 (0.0)	0 (0.0)	0 (0.0)	
	Z	Score 1	20 (100.0)	20 (100.0)	20 (100.0)	20 (100.0)	**…**
		Score 2	0 (0.0)	0 (0.0)	0 (0.0)	0 (0.0)	
Test of significance			**…**	**…**	**…**	**…**	**…**
Periodontal response	ZLS	Score 1	20 (100.0)	20 (100.0)	12 (60.0)	10 (50.0)	*P* < 0.001*
		Score 2	0 (0.0)	0 (0.0)	8 (40.0)	10 (50.0)	
	Z	Score 1	20 (100.0)	16 (80.0)	10 (50.0)	6 (30.0)	*P* < 0.001*
		Score 2	0 (0.0)	4 (20.0)	10 (50.0)	14 (70.0)	
Test of significance			**…**	*P* = 0.106	*P* = 0.525	*P* = 0.196	
Oral and general health	ZLS	Score 1	20 (100.0)	20 (100.0)	20 (100.0)	20 (100.0)	**…**
		Score 2	0 (0.0)	0 (0.0)	0 (0.0)	0 (0.0)	
	Z	Score 1	20 (100.0)	20 (100.0)	20 (100.0)	20 (100.0)	**…**
		Score 2	0 (0.0)	0 (0.0)	0 (0.0)	0 (0.0)	
Test of significance			**…**	**…**	**…**	**…**	

^∗^Score: 1 = clinically excellent/very good, 2 = clinically good, 3 = clinically sufficient/satisfactory, 4 = clinically unsatisfactory (but repairable), 5 = clinically poor (replacement necessary)

None of the 40 crowns exhibited fracture of restored teeth during the complete observation period and tooth integrity was scored as 1. Oral and general health was scored as 1 for all crowns in both groups during the 36-month follow-up period. Regarding periodontal response, all crowns of group ZLS were scored as 1 at baseline and 12-month follow-up visit. At the 24-month recall visit, eight crowns were scored as 2, and ten crowns were scored as 2 at the 36-month follow-up appointment. For group Z, the periodontal response was scored as 1 for all crowns at baseline and four crowns were scored as 2 in the 12-month follow-up appointment. After 2 years, ten crowns scored as 2 at 24 months, and 14 crowns scored as 2 at the 36-month follow-up appointments.

Periodontal probing depth (PPD) was reported to be increased at mesial and distal sites more than the facial one in the 36-month follow-up appointment with no statistically significant difference between both materials. A two-way ANOVA test was performed to analyze the effect of site and material on PPD separately and illustrated statistically no significant effect of changing the material (*P* = 0.186) while there was a statistically significant effect of changing the site (*P* = 0.001).

## Discussion

For the restoration to be successful, principles of tooth preparation; esthetic, function, and biological should be applied and respected [9]. In this study, FDI criteria were used to evaluate these principles. The vertical preparation was performed to test whether zirconia and ZLS ceramics could be used as esthetic crowns in very thin thickness [18]. The esthetic principles depend mainly on selecting the appropriate material, color selection, and the emergence profile of the restoration [36].

In this study, two high translucency ceramic materials were selected to mimic translucency and shade of natural teeth. Placing the margin sub-gingivally allowed achieving an optimal emergence profile. Regarding surface luster and staining, results showed that ZLS has higher surface luster and less staining than zirconia at 24- and 36-month follow-up. This could be attributed to the loss of zirconia surface glaze with subsequent surface roughness and also this was evident with recorded wear of opposing dentition which was apparent at the 36-month follow-up.

The incidence of complications, whether biological or mechanical, in the present study was not statistically significant [39]. One study [15] evaluated the clinical behavior of complete-coverage crowns and FDPs on teeth with vertical preparation without finish line. The sample included a total of 149 teeth that were prepared vertically without finish line with 0.5 mm prosthetic margin of zirconia. Two years after treatment, vertical preparation without finish line produced gingival thickening, margin stability, and optimal esthetics. Neither crowns nor FPDs presented any mechanical complications which coincided with the current study.

Although there was some controversy in the literature, as to whether or not, sub-gingivally placed restoration’s margin may or may not adversely affect the periodontal clinical parameters, it may reveal a detrimental effect on periodontal health if not managed well [40]. Gingival recession is associated with several factors, including gingival biotype (quality and quantity of keratinized gingival tissue), iatrogenesis during the dental preparation phase, chronic inflammation, and inadequate prosthetic marginal fit. Several studies have indicated that sub-gingival restorations with a conventional finish line are associated with periodontal inflammation and possible gingival recession [9, 12]. In a study to evaluate the influence of supra-gingival and sub-gingival margins on periodontal health, Dhanraj et al. reported that both margins similarly influence the periodontal health regarding plaque accumulation and gingival health status but an increase in pocket depth was observed with sub-gingival margins [41].

The present study obtained good gingival health outcomes in terms of pocket depth, inflammation, and bleeding on probing for both materials throughout the evaluation period. As already mentioned in the results, PPD was reported to be increased at mesial and distal sites more than the facial one in the 36-month follow-up appointment with no statistically significant difference between both materials. This finding could be related to patient’s difficulty to clean effectively the interproximal surfaces, as compared to the facial one. Furthermore, the restorative procedures—i.e., preparation, impression, and removal of luting agent’s excess are more difficult in these areas. It has been demonstrated that restorations with sub-gingival margins can contribute to plaque accumulation, especially in areas that are hard to be efficiently treated with scaling instruments [12].

Agustín-Panadero et al. [42] evaluated the clinical, mechanical, and biological behavior of posterior 3-unit FPDs placed on teeth prepared with BOPT. Forty participants received a 3-unit zirconia FPD in the posterior region of the mandible or maxilla. Twenty FPDs were placed on teeth prepared with BOPT (study group) and 20 on teeth with a horizontal chamfer finishing line (control group). After the 5-year follow-up, in the analysis of PPD, 26.3% of teeth in the control group had pockets of more than 3 mm in depth, whereas the BOPT group had only 10%. It was reported that posterior FPDs prepared by using BOPT had a good clinical response over a 5-year follow-up, with a low gingival index, a small increase in pocket depth, and a 100% marginal stability of the surrounding tissues. High survival rates after 5 years indicated that the technique produced predictable outcomes.

According to the technique used in this study, the biological width violation is practically not possible as the non-working tip of the special bur is calibrated so that not touching the first millimeter of the root where the connective tissue enters the cementum. Furthermore, the usage of a smaller tip permits a rotary curettage of the sulcus epithelium with little or no bleeding and a quicker healing [43, 44]. The interim crowns were used to direct the remodeling of gingiva through over-contouring or under-contouring to increase the gingival thickness [45]. The margins of restorations were designed to be with minimal thickness and extended only 0.5 to 1 mm sub-gingival to prevent violation of biological width and recession of gingiva.

The clinical success and survival of zirconia crowns fabricated with vertical margins were evaluated previously [8]. Results suggested that for zirconia crowns, vertical margins allowed clinical performance similar to that reported with other margin designs but with less invasive preparations which coincided with the current study. The periodontal response of periodontally healthy teeth restored using vertical preparation combined with a light rotary curettage (gingitage) was evaluated [33]. Results suggested that this protocol is a viable procedure; however, it was recommended to advocate longer follow-up studies.

Vertical preparation technique is complex and clinically more time-consuming. Moreover, situating the line of the prosthetic margin adequately is difficult because there is no definitive finish line exists. Also, there is a risk of uncontrolled invasion of the sulcus if performed by little experience dentist or technician. Excess cement was also difficult to be removed. The technique has not been backed by scientific evidence, and not enough research is available [46–48]. The relationship between the gingival biotype and the clinical outcome could not be established by the present study. The results of the present study may be considered preliminary, as bigger sample size and longer observational periods are probably needed to establish possible unidentified correlations between the examined parameters.

## Conclusions

Under the conditions of this study, the following conclusions were drawn:

1. Zirconia and ZLS could be used as a material for restoration of teeth prepared with vertical preparation technique.
2. Both ceramic materials achieved good esthetic results, promotes healthy and stable soft tissues with no mechanical complications after three years clinical evaluation.

## Data Availability

The datasets used and/or analyzed during the current study available from the corresponding author on reasonable request.

## Abbreviations

FDP: Fixed dental prosthesis
Y-TZP: Yttria-stabilized tetragonal zirconia polycrystal
CADCAM: Computer aided design-computer aided manufacturing
BOPT: Biologically oriented preparation technique
ZLS: Zirconia-reinforced lithium silicate
CEJ: Cemento-enamel junction
FDI: Federation Dentaire International
SD: Standard deviation
